# Peripheral T‐cell lymphoma of the oral cavity: A case report

**DOI:** 10.1002/cnr2.1751

**Published:** 2022-10-28

**Authors:** Amir Ali Asadi, Hamed Mahmoudi, Abbas Mofidi, Masoud Mortezazadeh, Alireza Hadizadeh

**Affiliations:** ^1^ Oral and Maxillofacial Surgery Department, Sina Hospital Tehran University of Medical Sciences Tehran Iran; ^2^ School of medicine Iran university of medical sciences Tehran Iran; ^3^ Internal Medicine Department Sina Hospital, Tehran University of Medical Sciences Tehran Iran; ^4^ School of medicine Tehran University of Medical Sciences Tehran Iran

**Keywords:** case report, non‐Hodgkin lymphoma, oral cavity, peripheral T‐cell lymphoma

## Abstract

**Background:**

Peripheral T‐Cell Lymphoma Not Otherwise Specified (PTCL‐NOS) is a rare type of non‐Hodgkin T‐cell lymphoma which frequently seen in immunocompromised individuals. It is estimated that only 2% of lymphomas are located on the buccal mucosa. In this case report, we present a 34‐year‐old male with a PTCL diagnosis.

**Case:**

A 34‐year‐old immune‐competent male presented with a buccal progressive ulcerated lesion. Histopathologic and immunohistochemical findings were compatible with PTCL‐NOS and classified as stage IIEA according to the Ann Arbor staging. The patient underwent chemotherapy followed by radiotherapy. He remained disease‐free after 12 months of follow‐up.

**Conclusion:**

Although lymphoma is uncommon in the oral cavity, physicians especially dentists in ordinary dental checkups should consider persistent progressive lesions as an important differential diagnosis of lymphoma.

## 1 INTRODUCTION

Lymphoma is the lymphatic system heterogeneous malignancies that are classified as Hodgkin's lymphoma (HL) and non‐Hodgkin's lymphoma (NHL).^1^ NHLs represent approximately 90% of all lymphomas and the second most common oral cavity malignancy after squamous cell carcinoma.^1^

Primary extranodal lymphoma represents 25%–40% of all NHL and about 2%–3% of them derive primarily from the oral cavity, which commonly affects the Waldeyer's ring.^2^ It is estimated that only 2% of these extranodal lymphomas are located on the buccal mucosa.^2^ Intraoral malignancies are about 2.5% of lymphoma cases.

Peripheral T‐cell lymphoma‐not otherwise specified (PTCL–NOS), are uncommon subtypes of NHL that arise due to inappropriate maturation of lymphoid lineage progenitor cells.^3^ It has poorly defined characteristics that make its diagnosis challenging. Based on few cases in the literature, it is associated with autoimmune disease, AIDS, infections with the human T‐cell lymphotropic virus, Epstein Barr virus and human herpesvirus.^1,3^ It should be noted that understanding its diagnosis and evaluation is difficult and limited because of its prevalence.^4^ Therefore, further studies are needed in this field to improve knowledge regarding its clinical presentation, histological evaluation, and management of patients.

## 2 CASE PRESENTATION

A 34‐year‐old man was admitted to the Maxillofacial Surgery department of Shariati hospital in 2021 with a chief complaint of facial asymmetry for 12 months.

The lesion started as a small painful lesion in the right buccal mucosa which progressed gradually and extended to the nasal cavity. Patients did not have any B symptoms including fever, weight loss, and night drenching.

His past medical history, psychosocial history, medication history, and family history were unremarkable except for the patient's habitual history of opium ingestion for 10 years.

After 3 months of local anti‐inflammatory and 14 days of antibiotics no expected therapeutic effect was seen.

Head and neck examinations revealed a firm, subcutaneous mass on the right side of the face. The mass was fixed and tender on palpation. Bilateral cervical lymph nodes were palpable.

Intraoral examination showed an ulcero‐proliferative lesion in the right buccal mucosa which had an ill‐defined and indurated margin and was covered with whitish pseudo‐membrane.

His blood investigations showed white blood cell count 3700/μl (neutrophils 87.0%, lymph 10.0%) (4.5 to 11.0 × 10^9^/L); hemoglobin 14.5 g/dl (14–17 g/dl); platelet count 196 × 10^9^/l (150 to 400 × 10^9^/L), ESR 47 mm/h (up to 10 mm/h), lactate dehydrogenase LDH 420 IU/L (150–500 U/L).

**FIGURE 1:**
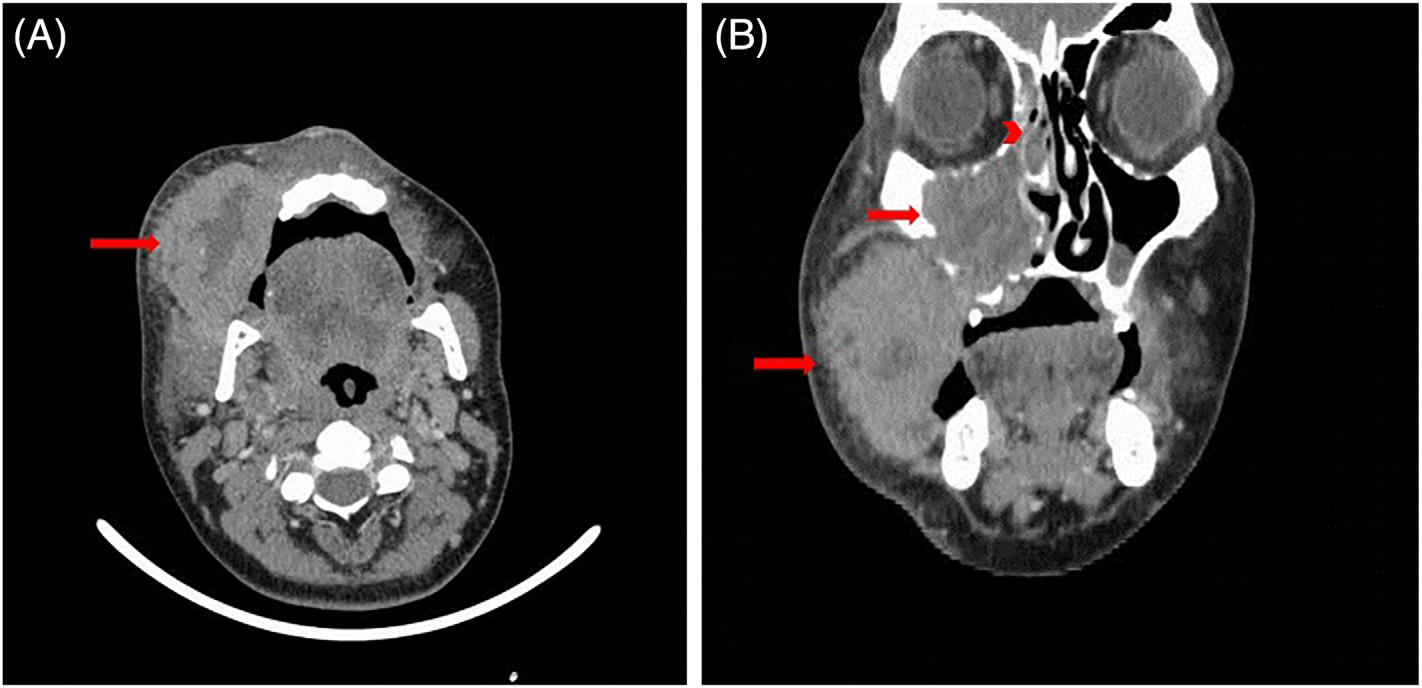
CT scan imaging showed Destructive and expansive mass with central necrosis with extension to maxillary and ethmoid sinuses (arrow: mass, notched arrow: maxillary sinus tumor extension, arrow head: ethmoid sinus tumor extension)

The patient underwent computed tomography (CT) scan of the oral cavity and neck. CT report was as follows: destructive and expansive soft tissue density with central necrosis in right maxillary sinus with extension to the nasal cavity and right ethmoid sinus with the destruction of the lateral wall of maxillary sinus and extension to cheek and buccal area. Secretions were also seen in the right sphenoid sinus. No intraorbital invasion was seen. Mild bilateral enlarged cervical lymph nodes were seen and the evidence in the CT was suggestive of reactive lymph nodes (Figure 1).

**FIGURE 2:**
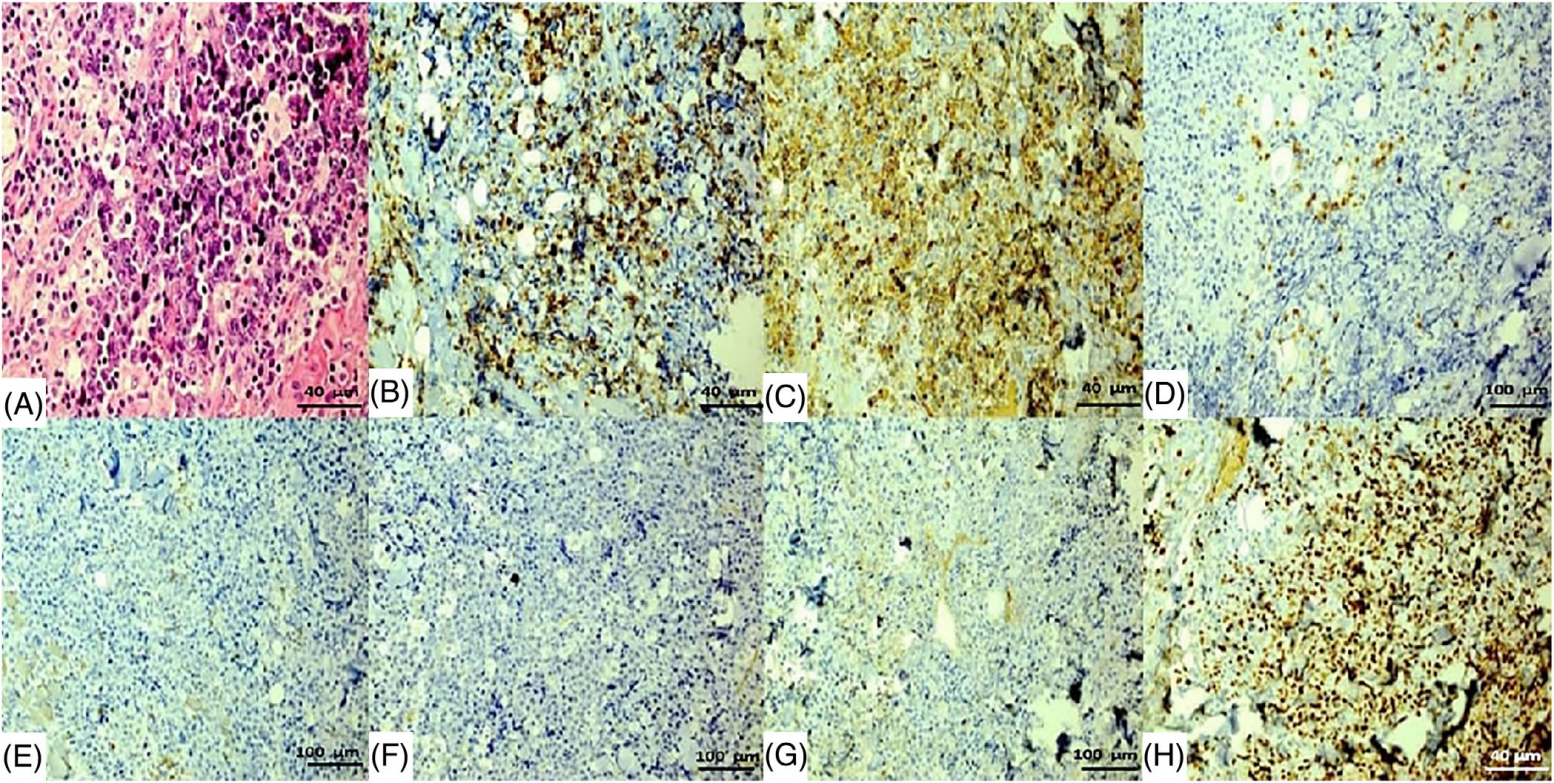
Pathology and IHC of peripheral T‐cell lymphoma, not otherwise specified (PTCL‐NOS), (A) H&E; the arrow indicates aggregation of T lymphocytes (×40), (B) LCA+ (×40), (C) CD3+ (×40), (D) CD7+ (×10), (E) CD5− (×10), (F) CD20− (×10), (G) CD30− (×10), (H) Ki67:70%–80% (×40)

Due to characteristic features of the imaging and clinical history of the patient, a biopsy from buccal mucosa was taken. Histopathologic and immunohistochemistry (IHC) findings were in favor of a rare diagnosis of Peripheral T‐cell lymphoma, not otherwise specified (PTCL‐NOS) (Figure 2).

IHC results were as follows: LCA+, CD3+, CD7+, CD5−, CD20−, CD30−, ALK1−, CD56−, and Ki67 was around 70%–80%.

Then a whole‐body bone and CT scan were performed for staging. Imaging findings did not reveal mediastinal or retroperitoneal lymphadenopathy or visceral involvement.

Bone marrow biopsy and cerebrospinal fluid cytology were negative for malignant cells.

Using the Ann Arbor staging, the patient was staged at the IIEA level of the disease.

After diagnosis, patient underwent chemotherapy followed by radiotherapy.

The chemotherapy regimen was CHOEP with the following drug doses:

Cyclophosphamide (Cytoxan) 750 mg/m^2^ IV on day 1.

Doxorubicin (Adriamycin) 50 mg/m^2^ IV on day 1.

Vincristine (Oncovin) 1.4 mg/m^2^ (max 2 mg) IV on day 1.

Etoposide (Vepesid) 100 mg/m^2^ IV on days 1–3.

Prednisone (Sterapred) 100 mg PO on days 1–5 and 6 cycles every 3 weeks.

This regimen was well tolerated without any major toxicity expect bone loss and osteopenia after the initiation of corticosteroid therapy which monitored by bone mineral density with dual X‐ray absorptiometry, Vitamin D and Calcium level in serum.^5^

As there was residue after chemotherapy, the patient received 3D conformal external beam radiotherapy for a total dose of 45 Gy in 25 fractions with five treatment fractions per week.

The patient achieved complete response and remains disease‐free without any major treatment‐related adverse effects after 12 months of follow‐up. After chemotherapy, according to not achieve a complete response to the treatment, we use a higher dose (45 Gy) in radiotherapy. Due to the ineffectiveness of chemotherapy and tumor relapse, the patient needs a long period of follow‐up.

According to previous studies the Median time from the end of treatment to relapse was 8 months (range 2–73) so long period follow‐up visits were scheduled.^6^

## 3 DISCUSSION

Less than 25% of NHL tumors present on extranodal sites that commonly involve the gastrointestinal tract and less than 25% of them involve the head and neck region.^2,3^ Based on previous reports, the oral cavity is involved only in 2% of cases.^1^ It is uncommon for NHL to primarily presents in the oral mucosa. Clinical presentation of PTCL‐NOS has extremely variable signs and symptoms that make its diagnosis difficult as clinicians should first role out other common diseases like SCC and salivary glands neoplasms.^7^

In this particular case, PTCL‐NOS not only involved buccal mucosa but also it extends to the nasal cavity, maxillary, sphenoid, and ethmoid sinus.

The etiology of PTCLs is unknown but some studies demonstrate the possibility of the association between exposure to specific viruses, such as the Epstein–Barr virus (EBV) and human T‐cell leukemia virus‐1 (HTLV‐1) with PTCLs.^8^

It is also mentioned in the literature that PTCL commonly occurs in patients with systemic conditions like AIDS, celiac disease, Crohn's disease, rheumatoid arthritis, congenital immunodeficiency, and Sjogren's syndrome.^1^, ^3^

However, our patient was not found to be suffering from any of these diseases and his test was negative for HIV Ab, HTLV‐1 Ab, EBV VCA‐IgM, and IgG.

A study of Kelly et al. showed evidence of an increased risk of NHL or multiple myeloma with exposure to lead, our case also had a history of oral opium ingestion which was highly suspected of contamination with lead.^9^ And in another study, Rana et al. also mentioned that tobacco exposure is known as another etiology of the malignancy.^10^

Based on previous studies, Immunohistochemistry can help with diagnosis and classification. On reviewing the literature, IHC showed a different pattern of cellular marker expression; in Gupta et al. study mentioned that peripheral T‐cell lymphoma not otherwise specified, includes T‐cell antigen expression and loss of one or more T‐cell‐associated antigens, such as CD2, CD3, CD5, or CD7. Around 65% of cases were CD4+ and 15% of cases were CD8+.^11^ PTCL was positive for Ki67 and T‐cell antigens especially CD3 and negative for B‐cell antigens like CD 20. HLA‐DR, CD25, and CD71 are usually expressed, while CD56, CD30, CD57, and TIA1, may not be seen. Adostinelli et al. study also mentioned that adult T‐cell acute lymphoblastic lymphoma or leukemia is associated with the human T‐cell leukemia virus‐1 (HTLV‐1) and is CD4/CD25+ another differential diagnosis is Anaplastic large‐cell lymphoma which is usually immune‐positive for CD30 and ALK1. New molecular genetic markers will also assist clinical investigators in designing improved therapies. According to their gene expression profile PTCL/NOS, with frequent extranodal and bone‐marrow involvement exhibit the upregulation of FN1, LAMB1, COL1A2, COL3A1, COL4A1, COL4A2, and COL12A1 in addition, it revealed the deregulation of genes involved in apoptosis such as MOAP1, ING3, GADD45A, and GADD45B and chemoresistance genes such as CYR61 and NNMT.^12^

In comparison to these studies, our case IHC was positive for CD3 and Ki67 and negative for CD20, also all the other IHC markers were negative in our case study.^2^, ^3^, ^13^, ^14^

It should be noted that there is a need for further studies to discover new cell markers in addition to current markers. Although it is noted in the literature that CD3 is correlated with a low response to treatment.^2^, ^10^

The Prognostic Index for PTCL is used for predicting overall survival by evaluating factors like age more than 60, elevated LDH level, bone marrow involvement, poor performance status, and Ki‐67 more than 80%. According to this scoring system, our patient scored 1 point which was identified as a low‐intermediate risk.^15^

Despite the PIT low‐intermediate risk, the overall 5‐year survival rate of PTCL patients is less than 20% and it is considered as an aggressive type of lymphoma.^16^

There are different treatment options for PTCL‐NOS like chemotherapy, radiotherapy, or its combination.

Chemotherapy with CHOP (cyclophosphamide, doxorubicin, vincristine, and prednisone) is considered the first‐line treatment.^3^, ^13^

According to Harris et al. study, an anthracycline‐containing regimen was not as effective as expected like the effect of an non‐anthracycline‐containing regimen in the treatment of diffuse large B‐cell lymphoma, the majority of patients with PTCL or NKTCL, other than ALCL, ALK positive.^17^ So further studies should be considered for the effect of anthracyclins.

Our patient underwent CHOP therapy followed by radiotherapy and his response to treatment was good, and he is disease free after in 12‐month follow up.

Therapeutic responses to this approach have been neither adequate nor durable and followed by poor prognosis, so in recent years newer agents like monoclonal antibodies such as alemtuzumab are under investigation for PTCL treatment.^18^

One of the strengths of our study is to highlight the importance of including PTCL in a differential diagnosis of oral malignant lesions even in patients who did not have mentioned factors that are associated with this disease.

## 4 CONCLUSION

In brief, our case highlighted the importance of including PTCL in a differential diagnosis of oral malignant lesions which present rarely in oral cavity, and early identification and prompt referral for Histopathology and immunohistochemistry investigation can lead to early diagnosis and appropriate treatment.

## AUTHOR CONTRIBUTIONS

**Amir Ali Asadi:** Writing – original draft (equal); writing – review and editing (equal). **Hamed Mahmoudi:** Writing – original draft (equal). **Abbas Mofidi:** Writing – original draft (equal). **Masoud Mortezazadeh:** Writing – original draft (equal); writing – review and editing (equal). **Alireza Hadizadeh:** Writing – original draft (equal); writing – review and editing (equal).

## CONFLICT OF INTEREST

The authors have stated explicitly that there are no conflicts of interest in connection with this article.

## ETHICS STATEMENT

In this study, we reported the retrograde standard treatment process of the patient. We maintained the patient's privacy, and his written consent was obtained. The participant has consented to the publication of this case report.

## Data Availability

The data that support the findings of this study are available from the corresponding author, [AM], upon reasonable request.
